# Robot-Assisted Diagnostic Laparoscopy: A Safe and Feasible Adjunct to the Management of Massive Spontaneous Pneumoperitoneum

**DOI:** 10.1155/2023/4722333

**Published:** 2023-03-09

**Authors:** Qingwen Kawaji, Sami Shoucair, Ali Darehzereshki, Alain Abdo

**Affiliations:** Department of Surgery, Medstar Franklin Square Medical Center, Baltimore, MD, USA

## Abstract

Pneumoperitoneum is the abnormal presence of free air in the peritoneal cavity. Oftentimes, it is a surgical emergency requiring exploratory laparotomy as most cases of pneumoperitoneum are due to perforated hollow viscus. However, not all pneumoperitoneum cases are surgical; nonsurgical pneumoperitoneum can arise from thoracic, abdominal, gynecologic, and other causes. We present a case of a 35-year-old male who developed a non-surgical pneumoperitoneum in the setting of drug overdose. The patient underwent robot-assisted diagnostic laparoscopy without findings of perforation or other pathology. Resolution of pneumoperitoneum was evidenced on follow-up computed tomography scan. This case emphasizes the importance of diagnostic laparoscopy in the setting of a confusing clinical picture and the feasibility of utilizing the robotic approach in hemodynamically stable patients.

## 1. Introduction

The abnormal collection of air in the peritoneal cavity can be due to a variety of causes and is not considered surgical in their entirety. In Mularski et al.'s systematic review of non-surgical pneumoperitoneum cases between 1970 and 1999, they found that in about 10% of patients, a non-surgical source is responsible for the free intra-abdominal air [1]. Furthermore, 61 (44%) of 139 patients with non-surgical pneumoperitoneum underwent surgery. However, these numbers are changing as the prevalence of complicated peptic ulcer disease decreases, and utilization of computed tomography (CT) scans increases. In their review of a database between 2000 and 2007, Kumar et al. found that only 41% of pneumoperitoneum cases had a perforated viscus [2]. Second to perforated viscus, the second most common cause of pneumoperitoneum was due to post-operative residual air (37%). Despite CT being the most sensitive and specific radiologic tool for evaluation of pneumoperitoneum, the incidence of non-surgical pneumoperitoneum is on the rise, and clinical correlation is critical.

Common surgical causes of pneumoperitoneum include perforation following peptic ulcer, diverticulitis, appendicitis, and endoscopy [2, 3]. Different causes of non-surgical pneumoperitoneum can be sub-categorized into four groups: thoracic, abdominal, gynecologic, and idiopathic/other. Thoracic causes are the most frequently reported etiologies for non-surgical pneumoperitoneum; they include intermittent positive pressure ventilation, barotrauma, cardiopulmonary resuscitation (CPR), bronchopulmonary fistula, pulmonary bleb rupture, adenotonsillectomy, pulmonary tuberculosis, bronchoscopy, coughing/Valsalva, and blunt trauma [1, 2]. Aside from previous surgery leading to retained air, other common abdominal etiologies of non-surgical pneumoperitoneum include pneumatosis cystoides intestinalis, endoscopic procedures, peritoneal dialysis, pneumo-cholecystitis, and diverticulosis [1, 4]. Gynecologic causes include coitus, vaginal insufflation and douching, pelvic inflammatory disease (PID), post-partum exercises, gynecologic exams and procedures, aquatic sports, and the rare case of Jacuzzi use in a patient with pre-existing vagino-peritoneal fistula [3, 5].

## 2. Case Presentation

**Figure 1:**
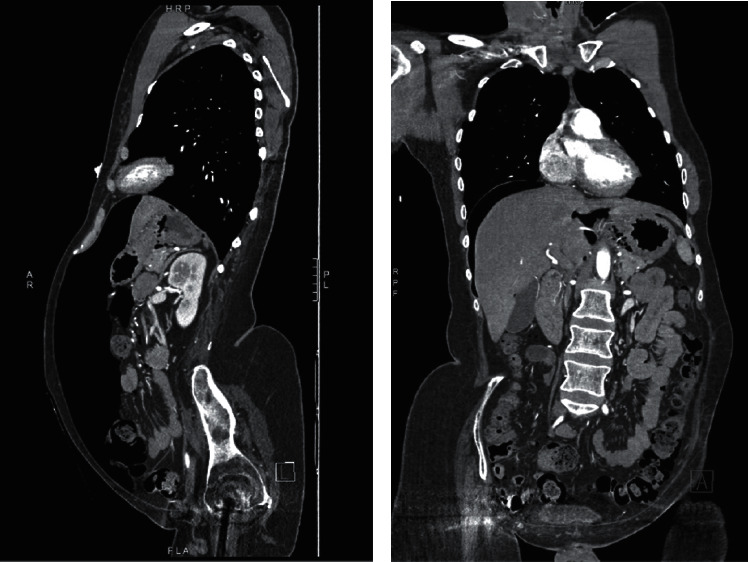
Computed tomography (CT) scan of the chest, abdomen, and pelvis; sagittal view (a) and coronal view (b).

**Figure 2:**
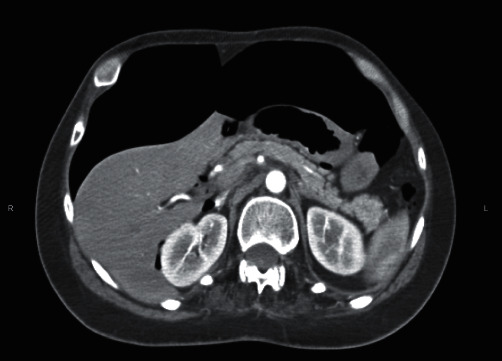
Computed tomography (CT) scan of the chest, abdomen, and pelvis (axial view).

A 35-year-old male with history of paraplegia due to T1–T6 spinal cord injury from a motor vehicle collision and heroin/cocaine use presented to the emergency department (ED) after a heroin overdose. Past medical history is also notable for peptic ulcer disease, multiple pressure ulcers status post debridement, and previous lower extremity Deep Venous Thrombosis (DVT) with Inferior Vena Cava (IVC) filter placement. Patient had snorted heroin and became unresponsive, for which the family called 911. When paramedics arrived at scene, patient was diaphoretic and hypotensive with a respiration rate of 4/minute. He responded to Narcan (2 mg of intranasal and 0.5 mg of intravenous doses). When patient initially arrived at the ED, he complained of shoulder pain, chest pain, back pain, and abdominal bloating. Hemodynamics were stable with an insignificant laboratory workup (Table 1). Physical examination was pertinent to abdominal distension and discomfort to palpation in all quadrants. There was no rebound tenderness or rigidity and no needle marks on the abdomen suggestive of injections. Patient's laboratory results in ED did not demonstrate any leukocytosis (white blood cell count= 6,300 cells/*μ*L) or lactic acidosis (1.4 mmol/L). His electrolytes were within normal limits (Table 1). CT scan of the chest, abdomen, and pelvis with intravenous contrast showed a large amount of free intraperitoneal air with small amounts of air dissecting upwards into the mediastinum (Figures 1 and 2).

Considering the patient's history of peptic ulcer disease (gastric ulcer) as well as his history of Non-Steroidal Anti-inflammatory Drug (NSAID) use for pain, the diagnosis highest on our differential was gastric ulcer perforation. The decision was made to perform robotic diagnostic laparoscopy due to concerns for delayed wound healing and infection in the setting of paraplegia and poor body hygiene.

**Figure 3:**
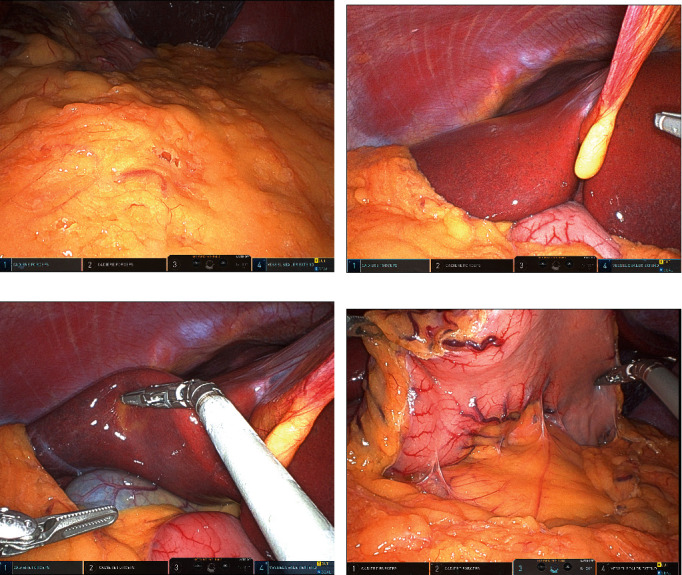
(a) Intraoperative images showing no bile, succus, or free fluid on abdominal entry. (b–d) Examination of all four quadrants as well as opening lesser sac were performed.

The da Vinci® Xi system (Intuitive Surgical, Inc., Sunnyvale, CA, USA) was docked after induction of general anesthesia. Intraoperatively, all intraperitoneal solid organs and hollow viscera were examined in all four quadrants in a systematic manner. The full length of the intra-peritoneal small bowel, colon, and rectum was evaluated. There was no sign of inflammation, ascites, biliary, or stool spillage. The gastrocolic ligament was dissected, and the retro-gastric space and first segment of the duodenum were inspected; no signs of inflammation or intragastric spillage were found. Upon cephalad retraction of the greater omentum and the transverse colon, the duodenojejunal junction was easily identified (Figure 3). Due to patient's small body habitus and minimal intra-abdominal/meso-transverse colon fat, we were able to inspect the retroperitoneal part of the duodenum through the transverse mesocolon with no signs of fluid collection or inflammation. The intraoperative exploration was completed by an upper gastro-esophageal endoscopy, which easily reached the third segment of the duodenum; no signs of perforation or inflammation were observed.

The patient did well during surgery, and his post-operative course was uncomplicated. A repeat CT scan of chest, abdomen, and pelvis with oral and rectal contrast on post-operative day 2 was performed as an adjunct to increase sensitivity for esophageal perforation and minimize any chance of a missed colonic/rectal/retroperitoneal perforation. All results were negative. Patient tolerated regular diet and was discharged on post-operative day 3. Finally, upon further investigation, the patient's father declared that the patient's uncle had performed vigorous CPR for a few minutes when the patient was unconscious, which has not been mentioned in the initial history.

## 3. Discussion

**Figure 4:**
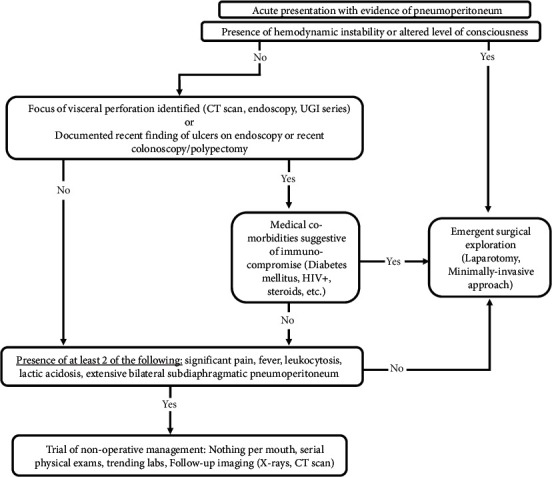
Suggested algorithm for approach to workup and management of spontaneous pneumoperitoneum.

True surgical pneumoperitoneum is often associated with obvious physical exam findings, such as abdominal pain, distension, and peritonitis [6]. Patients may present with fever, tachycardia, and leukocytosis. In absence of these findings, non-surgical causes should be highly considered [3, 7, 8]. However, many patients with non-surgical pneumoperitoneum are not completely asymptomatic, and some may have vital or laboratory abnormalities due to other co-existing conditions. In our case, the acute clinical complaints in the setting of extensive pneumoperitoneum in addition to a history of gastric ulcer/daily NSAIDs use persuaded the need for an emergent exploration. Our decision to explore robotically was driven by our concern for wound healing and the attending surgeon's substantial laparoscopic/robotic experience. We have proposed an algorithm for workup and treatment of pneumoperitoneum (Figure 4).

Robot-assisted diagnostic laparoscopy has recently been increasingly utilized in cases of emergency presentations. Kim et al. reported the case of a 17-year-old female who was found to have hemorrhage in the perirenal space in a trauma setting after a motor vehicle accident [9]. The patient was diagnosed with rupture of the ureteropelvic junction, which was safely repaired with a robot-assisted laparoscopic approach [10]. Other reports have been published in the literature advocating the feasibility of a minimally invasive approach in patient presenting to the ED if hemodynamic stability is maintained. In our reported case, the surgeon performing the procedure is fellowship-trained in minimally invasive surgery and comfortable with performing intra-abdominal acute care surgery on the robot.

## 4. Conclusion

Surgical exploration is a necessary part of the diagnostic workup of non-surgical pneumoperitoneum to avoid delay in treatment, particularly when the clinical picture is cloudy. In order to achieve the most with the least harm, we suggest the robotically assisted minimally invasive approach. Patient selection and indications for such an approach are not stringent and depend on the patient's hemodynamic stability and the surgeon's training and laparoscopic/robotic surgery experience. Regarding the benefit of the robotic-assisted approach compared with a purely laparoscopic diagnostic exploration, the former offers superior visualization with higher depth perception and increased degrees of motion with greater precision. Further studies comparing the difference in outcomes between the two approaches in diagnostic exploration are warranted in the future as the robotic approach becomes more widely accessible in institutions and surgeons more unanimously trained in both approaches.

## Figures and Tables

**Table 1:** Laboratory workup at the time of admission.

Vital signs	
Temperature (°C)	37
Heart rate (bpm)	79
Systolic Blood Pressure (SBP)/Diastolic Blood Pressure (DBP) (mmHg)	126/68
Respiratory Rate (RR) (breaths/minute)	19
Saturation (%)	100% on room air
Laboratory workup	
Sodium	141
Potassium	4.1
Chloride	109
Bicarbonate	31
Blood Urea Nitrogen (BUN)	20
Creatinine	1.1
Glucose	92
Lactic acid	1.4
White blood cell count	6.3
Hemoglobin	14.3
Hematocrit	44.3
Platelet count	257

Sodium, Potassium, Chloride. Bicarbonate: mmol/l (mmol/liter). BUN: mg/dl (milligram/deciliter). Creatinine: mg/dl (milligram/deciliter). Glucose: mg/dl (milligram/deciliter). Lactic acid: mmol/l (millimol/liter). White blood cell count: k/microliter (1000 cells/microliter). Hemoglobin: g/dl (gram/deciliter). Hematocrit: %. Platelet count: k/microliter (1000 cells/microliter).

## Data Availability

Data supporting this research article are available from the corresponding author or first author on reasonable request.
